# Viral Hepatitis and HIV Infection in Hemodialysis Patients

**DOI:** 10.5812/hepatmon.6959

**Published:** 2012-07-30

**Authors:** Arezoo Aghakhani, Mohammad Banifazl, Ali Eslamifar, Farrokhlagha Ahmadi, Amitis Ramezani

**Affiliations:** 1 Clinical Research Department, Pasteur Institute of Iran, Tehran, IR Iran; 2 Iranian Society for Support of Patients With Infectious Diseases, Tehran, IR Iran; 3 Nephrology Research Center, Tehran University of Medical Sciences, Tehran, IR Iran; 4 Pediatric Infectious Disease Research Center, Tehran University of Medical sciences, Tehran, IR Iran

**Keywords:** Hepatitis B Virus, Hepatitis C, Renal Dialysis

Dear Editor,

We greatly enjoyed reading the article by Zahedi et al. [1] about the prevalence of viral hepatitis and human immunodeficiency virus (HIV) infection in hemodialysis (HD) patients in South-East of Iran. They reported that hepatitis B surface antigen (HBsAg) and hepatitis C antibody (anti-HCV) were found in 7 % of cases individually. Anti-HIV and hepatitis D antibody (anti-HDV) were negative in all cases [1]. Patients on maintenance hemodialysis, potentially are prone to infection with parentally transmitted viral agents especially hepatitis B (HBV) and hepatitis C virus (HCV) due to impaired host immune response and multiple transfusion requirements .Viral hepatitis considered as a problem for HD patients because 1.9 % of all deaths among this population were related to the consequence of viral hepatitis [2][3]. The prevalence of HCV and HBV infection in hemodialysis patients is quite variable among different HD units in varying countries [4]. The mean prevalence of HCV in different HD facilities is 13.5 % with a range between 2.6 % - 22.9 % among countries [5]. The prevalence of HBsAg in hemodialysis patients is relatively low (< 10 %) in the developed countries however it’s higher (2 % - 20 %) within dialysis units in developing countries [6]. In Iran the prevalence of HBsAg and anti-HCV decreased from 3.8 % and 14.4 % in 1999 to 2.6 % and 4.5 % in 2006, respectively in HD units [7]. We carried out a similar study on 289 HD patients in Tehran. HBsAg, anti-HBs, anti-HCV, anti-HDV and anti-HIV were found in 2.8 %, 77.5 %, 3.1 %, 2.5 % and 0.34 % of patients, respectively. We detected less HBV and HCV infection, but more HDV and HIV infection in our cohort of patients than Zahedi et al. study [1][8]. In a systematic review by Alavian et al. [9] HBsAg positivity prevalence in general population of Tehran was 2.2 %, and we found slightly higher HBV prevalence in our HD patients. The prevalence of HCV infection varies widely between 5.5 % and 24 % among different Iranian populations [10] but we found less HCV sero positivity in our cases. These discrepancies in the rate of viral hepatitis infections in dialysis patients may reflect the diverse prevalence of these infections in different parts of country and within different dialysis units, different lengths of time on hemodialysis of the different populations, socioeconomic status, and size and composition of the study groups. In conclusion we suggest that further studies are needed to identify the trend of viral hepatitis infections in HD patients throughout the country. Effective strategies to reduce the prevalence and incidence of HCV and HBV infections among the dialysis patients should be implemented.
